# Mosquito bed net use and associated factors among pregnant women in Rwanda: a nationwide survey

**DOI:** 10.1186/s12884-023-05583-9

**Published:** 2023-06-06

**Authors:** Joseph Kawuki, Elorm Donkor, Ghislaine Gatasi, Lilian Nuwabaine

**Affiliations:** 1 Jockey Club School of Public Health and Primary Care, Faculty of Medicine, The Chinese University of Hong Kong, Hong Kong, China; 2 Key Laboratory of Environmental Medicine Engineering, School of Public Health, Southeast University, Nanjing, Jiangsu Province 210009 China; 3 School of Nursing and Midwifery, Aga Khan University, Kampala, Uganda

**Keywords:** Mosquito bed net, Usage, Insecticide-treated nets, Pregnant women, Rwanda

## Abstract

**Background:**

In malaria-endemic countries such as Rwanda, the appropriate use of mosquito bed nets is an effective intervention for malaria prevention. Despite being one of the demographics most impacted by malaria, there is a dearth of literature on the usage of mosquito bed nets by pregnant women in Rwanda. The study aimed to assess the prevalence and associated factors for mosquito bed net use among pregnant women in Rwanda.

**Methods:**

We used weighted data from the 2020 Rwanda Demographic and Health Survey of 870 pregnant women, and multistage stratified sampling was used to select participants. Multivariable logistic regression was conducted to determine the factors associated with mosquito bed net use, using SPSS (version 26).

**Results:**

Of the 870 pregnant women, 57.9% (95%CI: 54.6–61.1) used mosquito bed nets. However, 16.7% did not use bed nets among those owning bed nets. On one hand, older age (AOR = 1.59, 95%CI: 1.04–2.44), primary education (AOR = 1.18, 95%CI: 1.07–2.23), being married (AOR = 2.17, 95%CI: 1.43–3.20), being from Kigali region (AOR = 1.97, 95%CI: 1.19–3.91), partner’s education (AOR = 1.22, 95%CI: 1.13–3.41), having recently visited a health facility (AOR = 2.07, 95%CI: 1.35–3.18), and being in the third pregnancy trimester (AOR = 2.14, 95%CI: 1.44–3.18) were positively associated with mosquito bed net use. On the other hand, low wealth index (AOR = 0.13, 95%CI: 0.07–0.24), and being from Eastern region (AOR = 0.42, 95% CI: 0.26–0.66) had a negative association.

**Conclusions:**

About half of the pregnant women in Rwanda used mosquito bed nets and the usage was associated with various socio-demographics. There is a need for appropriate risk communication and continuous sensitisation to improve mosquito net use among pregnant women. Early antenatal care attendance and partner engagement in malaria prevention and mosquito net use, as well as consideration of household dynamics, are also crucial in improving not only mosquito net coverage but also utilization.

## Background

Malaria is a preventable and treatable parasitic infectious disease transmitted by the bite of a female anopheles mosquito [1]. An estimated 241 million malaria cases and 627,000 malaria deaths were reported globally in 2020, with the African region bearing the majority of the global malaria burden and thus accounting for 95% and 96% of the global cases and deaths respectively [2]. Most of the adverse effects associated with malaria infection are felt by children, immunocompromised individuals, and pregnant women [3]. Approximately thirty million pregnant women in Africa are estimated to be at risk of malaria infection annually, with possible severe adverse effects [3].

Malaria in pregnancy has been associated with significant morbidity and mortality for both the mother and the child as a result of poor maternal health and birth outcomes such as miscarriage, stillbirth, and intrauterine growth retardation [4, 5]. The World Health Organization (WHO) recommends the use of Long-Lasting Insecticidal bed Nets (LLINs), Intermittent Preventive Therapy in pregnancy (IPTp), early diagnosis and effective treatment as interventions for preventing and managing malaria during pregnancy [6]. Insecticide-treated net (ITN) use is beneficial for malaria prevention in areas of higher transmission and is thus recommended in addition to other preventive strategies [7]. Given the likelihood of low immunity among pregnant women, ITN use is vital even in areas of low transmission [4].

The targets set for malaria in pregnancy interventions have commonly not been met, especially the prompt use of ITN and IPTp, although challenges are yet to be overcome effectively [8, 9]. This gap in malaria prevention implies severe consequences for both the mother and the unborn child. As with other studies [4, 10], a prior study which examined the timing of malaria in pregnancy and its impact on infant growth and morbidity in Uganda shows that despite active screening, treatment and IPTp for the majority of women, malaria in pregnancy, particularly late pregnancy infection was associated with impaired infant growth [11]. As such, efficacious prevention of malaria, which includes prompt use of ITN, has the potential to reduce adverse consequences for mothers and infants [11, 12]. However, within the Sub-Saharan African region, of which Rwanda is a member state, aside from sociodemographic characteristics, the adoption and effectiveness of interventions in malaria prevention rely on a wide range of factors including awareness, attitude, and behaviour of target groups towards interventions which are in themselves shaped by social and cultural norms [5, 13–15].

Rwanda is a malaria-endemic country with the entire population considered at risk of malaria infection but more importantly pregnant women [16]. Although the country has high universal coverage of LLINs, there exist notable sociodemographic variations in the accessibility and utilization of bed nets in Rwanda [16, 17]. As such, rural areas with low literacy levels and wealth index were observed to have higher rates of non-usage of ITNs than urban areas [7, 18]. Moreover, a previous study indicated ITN use in rural areas of Rwanda to be 57% [19], a utilization rate that is extremely lower compared to the targeted 85% set by the Rwanda National malaria control program and previously recorded rates [16, 19]. Other factors previously associated with the utilization of bed nets include the number of household members, with lower use among households with five or more members; the number of bed nets present in households, with high numbers positively correlating with greater usage; as well as employment status, maternal educational attainment and antenatal (ANC) attendance and awareness of the importance bed net use [18, 20, 21].

Despite the available evidence of adverse pregnancy outcomes of malaria [4, 10], and differential ITN use in Rwanda, mosquito net use among pregnant women in the country has been barely investigated. Mosquito bed net use among pregnant women has been explored in other several malaria-endemic countries such as Uganda [21], Ethiopia [22], the Democratic Republic of Congo [23], and Malawi [24], among others. For Rwanda, only one study was conducted in Southern Rwanda and showed that although coverage of mosquito bed nets was high (84.1%), their utilization among pregnant women was lower (81.7%) than the national target of 85% [25]. However, this study considered only pregnant women in the southern region, with the uncertainty of what mosquito bed net use is in other regions of the country. Therefore, this study seeks to further examine the use of mosquito bed nets and associated factors among pregnant women in Rwanda, using the latest nationwide demographic health survey (DHS). Although WHO recommends ITNs as they are more effective than untreated nets [6], the latter also provides some form of protection against malaria [26] and this study considered both types of mosquito nets similar to previous studies [18, 25]. Understanding the possible determinants of mosquito bed net use among this special group is essential for informing policy and adoption of targeted interventions aimed at increasing the prompt use of ITN and thus curbing the adverse outcomes of malaria.

## Methods

### Study sampling and participants

This secondary data analysis used the 2019-20 Rwanda Demographic Survey (RDHS), which was a nationwide cross-sectional survey. Details of the study design, sampling and data collection have been reported elsewhere [17]. In brief, the RDHS used a two-stage sample design; with the first stage involving cluster selection consisting of enumeration areas (EAs), while the second stage involved systematic sampling of households in all the selected EAs leading to a total of 13,005 households [17]. The data used in this analysis were particularly from the household and the women’s questionnaires.

The data collection period for this survey was from November 2019 to July 2020, taking longer than expected due to the COVID-19 pandemic restrictions [17]. Eligible women for the RDHS interview were those aged 15–49 years, and either permanent residents of the selected households or visitors who stayed in the household the night before the survey. Out of the total 13,005 households that were selected for the survey, 12,951 were occupied and 12,949 were successfully interviewed leading to a 99.9% response rate [17]. In this analysis, we included only pregnant women interviewed during the survey, which were 870 out of the total 14,634 women in the whole survey [17].

### Variables

#### Dependent variables

The outcome variable is the use of a mosquito bed net (treated and untreated) a night before the survey among pregnant women, which was a binary outcome variable coded as yes or no, and was self-reported [17].

#### Explanatory variables

We included possible determinants of mosquito bed net use based on the available literature and data [14, 21–25]. Place of residence (categorized into rural and urban), region of residence (Kigali, South, West, East, North), and distance to a health facility (big problem, no big problem) were the community-level factors included. Household size (less than six, six and above family members), sex of household head (male, female), partner’s educational level and wealth index (categorized into five quintiles that ranged from the poorest to the richest quintile) were the household-level factors. In addition, we also included various individual-level factors; age (15–24, 25–34, 35 and above), working status (yes, no), parity (4 and less, above 4) educational level (no education, primary, secondary, tertiary), marital status (married, unmarried), religion (Catholic, Protestant, Adventist, others), health insurance (yes, no), visited a health facility in the last 6 months (yes, no), exposure to mass media (yes, no), pregnancy trimester (first, second, third), and visited by a fieldworker (yes, no). Wealth index was calculated by RDHS from information on household asset ownership using Principal Component Analysis [17]. Media exposure was when a woman had access to any of these; radio, newspapers and television. Marriage included those formally married, living together (cohabiting) or in a union, and the same applied to the classification of “partner”.

### Statistical analysis

We applied the DHS sample weights to account for the unequal probability sampling in different strata and ensure the representativeness of the study results [27, 28]. The Statistical Package for Social Sciences (SPSS) (version 26.0) software-complex samples package was used, incorporating the following variables in the analysis plan to account for the multistage sample design inherent in the RDHS dataset: individual sample weight, sample strata for sampling errors/design, and cluster number [17, 27]. We used frequency distributions to describe the background characteristics of the respondents; where frequencies and proportions/percentages for categorical dependent and explanatory variables have been presented. We then conducted bivariable logistic regression to assess the association of each explanatory variable with the dependent variable (mosquito bed net use), and we presented crude odds ratio (COR), 95% confidence interval (CI) and p-values. Explanatory variables found significant at a p-value < 0.25 were then included in the multivariable model, including those reported to have a significant association with mosquito bed net use in previous studies, regardless of their significance on bivariable analysis. Adjusted odds ratios (AOR), 95%CI and p-values were obtained and presented, with a statistical significance level set at p-value < 0.05. All explanatory variables in the model were assessed for multi-collinearity, which was considered present if a variable had a variance inflation factor (VIF) greater than 10 [29], but none of the variables had a VIF above 3. Missing data in explanatory variables were handled by a list-wise deletion in SPSS.

## Results

### Characteristics of participants

A total of 870 pregnant women were included in this analysis (Table 1). The majority were below 35 years of age (74.2%), working (66.1%), married (84.7%), with primary education (60%), and parity of below 4 (83.3%). Most of the respondents were rural residents (81.5%), covered with health insurance (89.1%), exposed to mass media (82.6%), from male-headed households (82%) of less than six household members (77.7%), visited a health facility in last 6 months (84.4%) and had no big problems with distance to a health facility (75.8%) (Table 1).

**Table 1:** Background characteristics of pregnant women and distribution of mosquito bed net use, as per the 2020 Rwanda Demographic Health Survey

Characteristics	Frequency (%), N = 870	Mosquito bed net use n(%), N = 503	P-value
**Age**			**0.005**
15–24	245(28.2)	122(24.2)	
25–34	400(46.0)	240(47.6)	
35 and above	224(25.8)	142(28.2)	
**Education level**			**< 0.001**
Tertiary	44(5.0)	36(7.1)	
Secondary	230(26.4)	146(29.0)	
Primary	522(60.0)	292(57.9)	
No education	74(8.6)	30(6.0)	
**Working status**			0.425
Working	575(66.1)	327(65.0)	
Not working	295(33.9)	176(35.0)	
**Parity**			0.854
Below 4	724(83.3)	418(83.1)	
4 and above	146(16.7)	85(16.9)	
**Marital status**			**< 0.001**
Married	736(84.7)	447(88.9)	
Unmarried	133(15.3)	56(11.1)	
**Religion**			0.362
Catholic	283(32.5)	175(34.8)	
Protestant	454(52.2)	253(50.3)	
Adventist	102(11.7)	56(11.1)	
Others	31(3.6)	19(3.8)	
**Health insurance**			**< 0.001**
Yes	774(89.1)	466(92.6)	
No	95(10.9)	37(7.4)	
**Wealth index**			**< 0.001**
Richest	190(21.8)	156(31.0)	
Richer	173(19.9)	117(23.2)	
Middle	178(20.5)	98(19.4)	
Poorer	174(20.1)	84(16.7)	
Poorest	154(17.7)	49(9.7)	
**Residence**			**< 0.001**
Urban	161(18.5)	123(24.5)	
Rural	708(81.5)	380(75.5)	
**Region**			**< 0.001**
Kigali	110(12.6)	95(18.9)	
West	199(22.9)	116(23.1)	
East	222(25.5)	95(18.9)	
North	131(15.1)	75(14.9)	
South	208(23.9)	122(24.3)	
**Household size**			0.410
Less than 6	676(77.7)	396(78.7)	
6 and above	194(22.3)	107(21.3)	
**Sex of household head**			0.181
Male	713(82.0)	421(83.5)	
Female	156(18.0)	83(16.5)	
**Partner’s education** ^**a**^			**< 0.001**
Tertiary	62(7.2)	52(11.6)	
Secondary	120(13.9)	80(17.9)	
Primary	460(52.9)	268(60.0)	
No education	94(10.8)	47(10.5)	
**Exposure to media**			**0.005**
Yes	718(82.6)	432(85.7)	
No	151(17.4)	72(14.3)	
**Distance to a health facility**			0.151
No big problem	659(75.8)	390(77.5)	
Big problem	210(24.2)	113(22.5)	
**Visited a health facility in the last 6 months**			**< 0.001**
Yes	734(84.4)	454(90.1)	
No	136(15.6)	50(9.9)	
**Pregnancy trimester**			**< 0.001**
First	256(29.5)	129(25.6)	
Second	326(37.5)	183(36.4)	
Third	287(33.0)	191(38.0)	
**Visited by fieldworker in 6 months**			0.265
Yes	358(41.2)	216(42.9)	
No	511(58.8)	287(57.1)	

a = 133 missing values

The use of mosquito bed nets was significantly higher among pregnant women from rural areas (75.5%), those aged 25–34 years (47.6%), those with primary education (57.9%), the married (88.9%), those with health insurance (92.6), and exposed to mass media (85.7%). Pregnant women in the third trimester (38.0%), who had visited a health facility in the last 6 months (90.1%), in the richest wealth quintile (31.0%), from the southern region (24.3%) and whose partners had primary education (60.0%) also had higher rates of mosquito bed nets, Table 1.

Of the 870 pregnant women, 503 (57.9%, 95%CI: 54.6–61.1) used mosquito bed nets, of which 97% used insecticide-treated mosquito bed nets while 3% used untreated mosquito bed nets. Notably, 16.7% did not use mosquito bed nets out of the 604 pregnant women owning a bed net (Table 2).

**Table 2:** Ownership and use of mosquito bed nets

**Mosquito bed net ownership**	**Frequency (%), N = 870**
Yes	604 (69.5)
No	265 (30.5)
**Slept under mosquito bed net all respondents**	**Frequency (%), N = 870**
Yes	**503 (57.9), (95%CI: 54.6–61.1)**
No	366 (42.1)
**Slept under bed net among those with nets**	**Frequency (%), N = 604**
Yes	503 (83.3)
No	101 (16.7)
**Type of bed net slept under**	**Frequency (%), N = 503**
Treated net	488 (97.0)
Untreated net	15 (3.0)

### Factors associated with the use of mosquito bed nets

Results of the bivariable analysis are detailed in Table 3 with factors individually associated with bed net use highlighted. In the final multiple logistic regression model, the factors found significantly associated with mosquito bed net use were; age, educational level, marital status, wealth index, region, partner’s education, visited a health facility in the last 6 months, and pregnancy trimester (Table 3).

**Table 3:** Factors associated with mosquito bed net use among pregnant women, as per the 2020 Rwanda Demographic Health Survey

Variable	Crude odds ratio, COR (95% CI)	p-value*	Adjusted odds ratio, AOR (95% CI)	p-value**
**Age**		**0.013**		**0.012**
15–24	1		1	
25–34	1.51(1.09–2.10)		1.24(0.86–1.79)	
35 and above	1.74(1.17–2.60)		**1.59(1.04–2.44)**	
**Education level**		**< 0.001**		**0.030**
No education	1		1	
Primary	1.92(1.12–3.28)		**1.18(1.07–2.23)**	
Secondary	2.64(1.45–4.83)		0.10(0.48–2.09)	
Tertiary	6.52(2.48–17.15)		0.89(0.26–3.02)	
**Working status**		0.448		0.909
Not working	1		1	
Working	0.88(0.64–1.22)		0.98(0.67–1.42)	
**Parity**		0.886		0.746
Below 4	1		1	
4 and above	1.03(0.70–1.52)		0.90(0.49–1.67)	
**Marital status**		**< 0.001**		**0.003**
Unmarried	1		1	
Married	2.14(1.43–3.20)		**2.17(1.43–3.20)**	
**Religion**		0.420		0.416
Catholic	1		1	
Protestant	0.78(0.56–1.10)		0.77(0.52–1.13)	
Adventist	0.77(0.47–1.24)		0.71(0.41–1.22)	
Others	1.04(0.50–2.17)		1.05(0.48–2.29)	
**Health insurance**		**< 0.001**		0.340
No	1		1	
Yes	2.36(1.50–3.71)		1.30(0.76–2.23)	
**Wealth index**				
Richest	1	**< 0.001**	1	**< 0.001**
Richer	0.45(0.26–0.77)		**0.52(0.30–0.91)**	
Middle	0.27(0.16–0.45)		**0.34(0.19–0.61)**	
Poorer	0.20(0.12–0.34)		**0.25(0.14–0.44)**	
Poorest	0.10(0.06–0.18)		**0.13(0.07–0.24)**	
**Residence**		**< 0.001**		0.449
Rural	1		1	
Urban	2.82(1.85–4.31)		0.83(0.51–1.34)	
**Region**		**< 0.001**		**< 0.001**
South	1		1	
North	0.93(0.55–1.44)		0.91(0.54–1.52)	
East	0.53(0.34–0.80)		**0.42(0.26–0.66)**	
West	0.99(0.61–1.59)		0.76(0.45–1.28)	
Kigali	4.42(2.34–8.34)		**1.97(1.19–3.91)**	
**Household size**		0.432		0.110
Less than 6	1		1	
6 and above	0.87(0.61–1.24)		0.70(0.46–1.08)	
**Sex of household head**		**0.193**		0.374
Male	1		1	
Female	0.78(0.54–1.13)		0.83(0.56–1.24)	
**Partner’s education**		**< 0.001**		**0.014**
No education	1		1	
Primary	1.39(0.85–2.28)		1.02(0.58–1.79)	
Secondary	1.95(1.08–3.53)		0.90(0.43–1.86)	
Tertiary	4.90(2.16–11.13)		**1.22(1.13–3.41)**	
**Exposure to media**		**0.007**		0.811
No	1		1	
Yes	1.67(1.15–2.43)		0.95(0.62–1.45)	
**Distance to a health facility**		**0.161**		0.622
No big problem	1		1	
Big problem	0.79(0.57–1.10)		1.10(0.75–1.60)	
**Visited a health facility in the last 6 months**		**< 0.001**		**0.001**
No	1		1	
Yes	2.82(1.89–4.19)		**2.07(1.35–3.18)**	
**Pregnancy trimester**		**0.002**		**0.001**
First	1		1	
Second	1.25(0.85–1.83)		1.35(0.89–2.04)	
Third	1.97(1.34–2.87)		**2.14(1.44–3.18)**	
**Visited by fieldworker in 6 months**		0.258		0.264
No	1		1	
Yes	1.18(0.89–1.57)		1.20(0.87–1.67)	

**Bold** = significant, * significant at 0.25, ** significant at 0.05

Compared to women of 15–24 years, those of 35 years and above (AOR = 1.59, 95%CI: 1.04–2.44) had more odds of using mosquito bed nets, same as those with primary education (AOR = 1.18,95%CI: 1.07–2.23) who had more odds compared to their counterparts with no education. Married pregnant women (AOR = 2.17, 95%CI: 1.43–3.20) also had more odds of using bed nets compared to the unmarried. Low wealth index was associated with lower odds of mosquito net use, where pregnant women in the richer (AOR = 0.52, 95%CI: 0.30–0.91), middle (AOR = 0.34, 95%CI: 0.19–0.61), poor (AOR = 0.25, 95%CI: 0.14–0.44) and poorest (AOR = 0.13, 95%CI: 0.07–0.24) quintiles were 48%, 66%, 75%, and 87% less likely to use bed nets, respectively, compared to their fellows in the richest wealth quintile. Compared to pregnant women in the Southern region, those in Kigali (AOR = 1.97, 95%CI: 1.19–3.91) had more odds of using mosquito bed nets, unlike those in the Eastern region (AOR = 0.42, 95%CI: 0.26–0.66) who had less odds. Moreover, partner’s education was positively associated with mosquito bed net use, where pregnant women with partners having tertiary education (AOR = 1.22, 95%CI: 1.13–3.41) had more odds of using bed nets compared to those with partners having no education. Pregnant women who visited a health facility in the last 6 months (AOR = 2.07, 95%CI: 1.35–3.18) had more odds of using bed nets compared to those who did not, and the same applied to those in the third trimester (AOR = 2.14, 95%CI: 1.44–3.18) who had more odds of using bed nets compared to their counterparts in the first trimester (Table 3).

## Discussion

The current study was conducted to evaluate the prevalence and factors associated with mosquito bed net use among pregnant women in Rwanda. Despite 69.5% of pregnant women reporting owning a mosquito net, only 57.9% attested to having slept under it. This usage is lower than what was observed in studies conducted in other malaria-endemic countries such as the Democratic Republic of Congo (71.4%) [23], Mozambique (68.4%) [30], and Ghana (61%) [31], but it is also higher than others like Malawi (53%) [24], Uganda (35%) [21] and Ethiopia (39.9%) [22]. It also represents a decrease in usage from prior estimates (69%) reported in the 2017 Rwanda Malaria Indicator Survey (RMIS) [32]. Nevertheless, a logical explanation would be that different countries have different risk levels of malaria due to differences in climate and geography, and so have different malaria control strategies. Moreover, the season in which data was collected is a possible explanation, since mosquito net use tends to be higher during malaria transmission peaks, which usually correspond with wet/rainy seasons [33]. This underlines the significance of ensuring ongoing bed net availability, prompt procurement, and education/ sensitisation on the proper use of mosquito bed nets. According to prior research, a major barrier to mosquito bed net use remains the lack of access, and therefore, an accessible supply significantly improves their use [34, 35]. Notably, results indicate that despite insecticide-treated mosquito nets being provided for free at ANC visits in Rwanda [16, 36], some (3%) still use untreated nets which are less effective compared to treated ones. The reason for this observation is unclear but may be partly due to the unpleasant odour of treated nets (to some people), and the preference for fancy-designed bed nets which are in most cases untreated [26]. Such concerns should be considered, explored and addressed to ensure the maximum utilization of ITNs among pregnant women in Rwanda.

In addition, our study findings showed substantial variations in mosquito bed net use by socioeconomic and demographic factors. Educational attainments of both women and their partners were found to be significant predictors in the use of bed nets. Specifically, pregnant women with primary education had higher odds of utilizing bed nets than those with no education, just like pregnant women with partners who had higher education had greater odds of using them over those who didn’t. This finding corroborates prior research which also reported a positive impact of education on mosquito bed net use [30, 37, 38]. This is not surprising given that individuals with a higher level of education are also more likely to seek additional learning. Lower levels of education might, thus, lead to a lack of comprehension of the rationale for the necessity of adopting healthy behaviour and how that relates to disease prevention, and in this particular instance, the use of mosquito bed nets and how it prevents malaria, as well as how to properly hang and set them up. Without a doubt, this difficulty might be circumvented by considering the target demographic when imparting knowledge on mosquito bed net use [39].

Our study also found a significant relationship between mosquito bed net use and the pregnancy trimester. When compared to women in the first trimester, third-trimester pregnant women were twice as likely to use bed nets, and the usage of bed nets was influenced by recent visits to a medical facility. This is similar to what was reported in similar studies from Ethiopia and Ghana where second and third-trimester pregnant women had significantly more odds of using ITNs than their first-trimester counterparts [22, 40]. The likely explanation is that early in pregnancy, particularly compared to later in pregnancy, women may not have attended a health facility for the opportunity to be taught or reminded of the necessity of sleeping under a mosquito net. Moreover, antenatal care and immunization visits are some of the most common sources of free mosquito bed nets in Rwanda [16, 36] and other countries like Cameroon [41]. In corroboration, our study findings indicated higher odds of mosquito net use among pregnant women with a recent visit to a health facility. This further stresses the need for early and consistent antenatal care attendance as well as the use of such contacts with health personnel for continuous health education and counselling at every stage of pregnancy.

Pregnant women in Kigali, the capital and largest city of Rwanda, had a higher likelihood of utilizing mosquito bed nets than those from the Southern region. The observation aligns with findings from a previous review that reported mosquito net use to vary with region and area of residence [38]. This can, however, be explained by the regional difference in malaria risk in Rwanda owing to the differential distribution of factors that favour mosquito breeding such as climatic conditions and population density [16, 36]. In this regard, customised malaria prevention strategies have been adopted in Rwanda for example the use of larvicides in the Eastern and Southern provinces [16], which may explain the low mosquito bed net usage in such regions. Nonetheless, given the likelihood of low immunity among pregnant women, mosquito net use remains indispensable even in such areas of seemingly low transmission risk [4]. This implies appropriate communication and sensitisation with emphasis on the possible malaria risk among pregnant women to address such risk compensation misconceptions regarding mosquito net use, especially as recent evidence shows that the higher malaria incidence is found in the Southern districts of Rwanda [36].

It was observed that using a mosquito bed net was less likely among unmarried, younger pregnant women and those from lower-income households. These findings are in agreement with previous studies. Two studies earlier both established lower rates of mosquito net use among younger pregnant women compared to older ones [24, 42], which, arguably, might be because older women are more likely to have prior pregnancy experiences or even adverse consequences of malaria in pregnancy, thus more likely to understand the importance of mosquito bed net use. Likewise, as previously determined by *Wagbatsoma et al.*. and *Dun-Dery et al.*, more married than unmarried women slept under ITNs [40, 43]. Household wealth index has also previously been identified as a positive predictor of ITN usage among pregnant women in Nigeria [44]. This suggests that previous pregnancy (a proxy for older age), spousal support, and a stable financial status may all play important roles in bed net use. It, thus, implies it would be beneficial to consider age, previous pregnancy experience, marital status and household income when designing malaria prevention as well as mosquito net use programs and policies. Having targeted intervention for these specific population groups would be vital for successful campaigns to maximize not only coverage but also usage.

### Strength and limitations

We used a weighted dataset of the most recent nationwide DHS, implying that our findings can be generalised to all pregnant women in Rwanda. Moreover, DHS follow standardized high-quality data collection protocols, with larger sample sizes, making our findings comparable to other countries.

Nonetheless, the present study has some weaknesses, just like other previous comparable investigations. There is a risk of information and recall bias due to the social desirability of mosquito bed net use and the fact that most of the data were self-reported, with no record justification. This could have affected the true estimation of mosquito bed net use among this population group. The lack of accurate information on malaria prevention and control in different countries limited a more in-depth comparison of the observed correlates with other countries/regions. Moreover, the use of mosquito bed nets was only assessed the night before the survey; therefore, it may not accurately reflect the trend of usage over time. In addition, the cross-sectional design of the study limits the determination of a causal relationship but rather only association. Nonetheless, the study provides valuable information on mosquito net use and its associates among this special group, despite the limitations.

## Conclusions

This study demonstrated that the use of mosquito bed nets among pregnant women in Rwanda still needs to level up. We found several socio-demographic factors associated with the use of mosquito bed nets, which included age, educational level, marital status, wealth index, region, partner’s education, visit to a health facility, and pregnancy trimester.

The study highlighted the need for appropriate risk communication and sensitisation among pregnant women to address the low usage of mosquito bed nets in regions/areas with alternative prevention strategies/ low transmission risk. The need for early antenatal care attendance, partners’ engagement in malaria prevention and mosquito net use, as well as an accessible supply of bed nets, is also highlighted. In addition, consideration of household dynamics such as wealth index, and marital status amongst others is also crucial for improving not only mosquito net coverage but also utilization.

## Data Availability

The data set used is openly available upon permission from the MEASURE DHS website (URL: https://www.dhsprogram.com/data/available-datasets.cfm). However, authors are not authorized to share this data set with the public but anyone interested in the data set can seek it with written permission from the MEASURE DHS website (URL: https://www.dhsprogram.com/data/available-datasets.cfm).

## Abbreviations

ANC: Antenatal care
AOR: Adjusted Odds Ratio
CI: Confidence Interval
COR: Crude Odds Ratio
DHS: Demographic Health Survey
EA: Enumeration area
IPTp: Intermittent Preventive Therapy in Pregnancy
ITN: Insecticide-treated net
LLINs: Long-Lasting Insecticidal bed Nets
RDHS: Rwanda Demographic Health Survey
RMIS: Rwanda Malaria Indicator Survey
SPSS: Statistical Package for Social Science
SSA: Sub-Saharan Africa
VIF: Variance inflation factor
